# Challenges of organizing pediatric anesthesia in low and middle-income countries

**DOI:** 10.1016/j.bjane.2024.844525

**Published:** 2024-06-19

**Authors:** Anelise Schifino Wolmeister, Tom G. Hansen, Thomas Engelhardt

**Affiliations:** a Montreal Children's Hospital, Department of Anesthesia, Montreal, Canada; b Hospital de Clínicas de Porto Alegre, Departamento de Anestesia e Medicina Perioperatória, Porto Alegre, RS, Brazil; c Akershus University Hospital, Department of Anesthesia & Intensive Care, Lørenskog, Norway; d University of Oslo, Institute of Clinical Medicine, Oslo, Norway

“*Children have the right to enjoy the highest attainable standard of health. Specialist pediatric anesthesia care should be provided for all children. Particularly children aged less than three years should be treated by experienced staff that follows continuous education, regular training and updates to maintain their competencies. Children who have significant comorbidity and those who require highly specialized or major surgery benefit from specialized anesthetic care in dedicated pediatric centers.*”

*The Safe Anesthesia For Every Tot initiative*

Pediatric anesthetists are recognized as highly specialized professionals, tasked with the complex responsibility of taking care of a vulnerable population. This expertise requires a delicate balance between scientific mastery, precision, and steady commitment to the safety of the young ones. However, the dynamics of this profession, and the pursuit of safety within, can vary significantly across the globe. From the individual level – regarding standards, requirements, and training opportunities – to the environmental characteristics – settings and resources – there is a significant difference in the quality of the pediatric anesthesia provided worldwide. Achieving and maintaining institutional competencies as delineated by the Safe Anesthesia for Every Child (Safetots) initiative (www.safetots.org)^1^ (Table 1), poses significant challenges in Low- and Middle-Income (LMIC) countries such as Brazil and the wider South America.

**Table 1:** Institutional competence (adapted from safetots.org).

**W**ho	• Provider: Anesthesiologist with special training in pediatric anesthesia. Residents and fellows are supervised in a 1:1 manner by experienced staff
	• Patient: Children < 3 years, ASA ≥ III, underlying congenital and metabolic diseases, undergoing major or complex surgery
**W**here	In pediatric hospitals or in general hospitals with dedicated pediatric areas. Referral pathways to resourced multidisciplinary pediatric environments
**W**hat	Highly specialized/ major surgery (cardiac, thoracic, major visceral, major orthopedic, neurosurgical, burns and craniofacial procedures), complex comorbidities and critically ill children must have specialized anesthetic care in dedicated pediatric centers
**W**hen	Balance between increased perioperative risks for newborns and infants and impact of procedure delay.
Ho**W**	High Quality and Safe Anesthesia Care. Expertise in pediatric anesthetic techniques guaranteeing optimal care for all children in all situations

According to The Lancet Commission on Global Surgery, the distribution of the specialist surgical workforce is measured by the density of specialist surgeons, anesthetists, and obstetricians per 100,000 population and correlates with specific health outcomes. For example, countries with a higher density of providers per 100,000 population have lower maternal mortality. In 2015, the recommendation of specialist surgeons, anesthetists, and obstetricians was 20 per 100,000 population with the goal of 20 to 40 by 2030. Not surprisingly, shortages and maldistributions of these professionals are seen within and among LMIC leading to serious inequity. Specialists are frequently concentrated in urban areas with higher surgical infrastructure and working in better-equipped tertiary care centers, leaving the rural areas neglected.^2^

Following this same pattern, the Brazilian reality is illustrated by the 2023 demographic data of 13.7 anesthesiologists per 100,000 population varying from 4.2 in the province of Acre to 30.6 in the Federal District, and 68.5 in the south and southeast of the country.^3^ This is in stark contrast to the USA for example where an average of 5.4 pediatric anesthesiologists per 100,000 are available for children alone.^4^

Training a new anesthesia workforce takes time and is unlikely to be achieved by 2030. In High-Income Countries (HICs) the training for anesthesia varies between 4 to 5 years and the requirements include pediatric anesthesia exposure in the early years of training under the direct supervision of a pediatric anesthetist. Most European countries have an additional organized society for pediatric anesthetists committed to continuing education in pediatric anesthesia. The regular anesthesia residency program in Brazil is 3 years and residents only rotate in pediatric anesthesia during their last year of residency. Currently, five centers in Brazil offer a four-year training in pediatric anesthesia. Pediatric anesthesia as a structured and recognized subspecialty, however, is not organized and hence has limited national representation. A direct consequence is that there are no public available data on the pediatric anesthesia workforce in Brazil.

Availability of pediatric anesthesia expertise is critical and affects patient outcomes. In 2017, the Anaesthesia PRactice In Children Observational Trial (APRICOT) study analyzed the incidence of severe critical events in children undergoing general anesthesia in Europe, highlighting key concepts of the population's vulnerability and urging targeted education and strategies to improve quality in pediatric anesthesia.^5^ The very same observations also hold true in LMICs. A multicenter observational cohort in South Africa evaluated the Severe Anesthesia-Related Critical Incidents (SARCIs). They reported SARCI of almost 16% in a population composed of two-thirds healthy patients of which more than half underwent minor surgery. Factors such as young age, urgency of the procedure, severity of the surgery, and level of hospital independently increased the risk for SARCI. Compared with medium to high-income countries the SARCI events were 3 times higher, and the incidence of Perioperative Cardiac Arrests (POCA) was 10 times greater.

These results reinforce the notion that pediatric anesthesia care needs to be provided by staff trained in pediatric anesthesia in particular when it involves infants and neonates.^6^ The latter is further underlined by the NECTARINE study.^7^ Safety in pediatric anesthesia needs to be addressed urgently, that is evident in the article “Access to Safe Pediatric Anesthesia in LMICs-The Problem Is Clear; It Is Time to Solve It!”.^8^ Data from a tertiary teaching hospital in Brazil reported at least twice as high incidence of anesthesia-related cardiac arrest (2.81 per 10,000) when compared with HIC (from 0.14 to 1.4 per 10,000). Young age, an American Society of Anesthesiologists (ASA) physical status greater than III as well as difficulties with airway management are the greatest causes of anesthesia-related cardiac arrest and illustrate the need for trained pediatric anesthesia staff with appropriate pediatric resources.^9^

## Who and when

Another huge barrier to the improvement of quality and safety in Brazil and South American LMICs is the model of healthcare delivery: The role of the physician is often the central point of care underestimating the real requirement of other well-trained healthcare professionals. The practice of anesthesia is no different, the physician plays a pivotal role in decision-making and the care provision is delivered in a system based on hierarchy. Opposed to that, in high-income countries, the healthcare system has evolved to embrace a multidisciplinary approach and collaborative teamwork, where responsibilities are shared among professionals such as anesthesiologists, nurses, and respiratory specialists.

This comprehensive model of care ensures safety, especially in the context of the pediatric population, where the tasks are more complex and time-critical due to the higher fragility of the patients. Even though Europe and North America have different structured healthcare systems, the overall practice of anesthesia relies heavily on the expertise of the non-medical personnel. Anesthesia is either provided directly by a physician or supervised by a physician in most of the European countries while in the US nurse anesthetists may provide direct care medical guidance. In both, trained nurse anesthetists or anesthesia technicians work together with the physicians, assuming different levels of independence, and focusing on providing excellent clinical care.^10^

This huge discrepancy among the anesthesia workforce worldwide is well documented by the World Federation of Societies of Anesthesiologists (WFSA). For example, the workforce in the US has more than 33,000 nurse anesthesia providers as well as almost 2,000 other anesthesia providers while in Brazil there are no nurse anesthesia providers or other supporting professionals.^11^

Certainly, the transition to a decentered and multidisciplinary system is a difficult task, yet it is based on a fundamental principle: shared expertise leads to improved safety and quality of care.

## How

There is no doubt that pediatric anesthesia care for sicker and younger children at a tertiary specialized pediatric center improves patient outcomes. Unfortunately, there is a mismatch between individual patient needs and available clinical resources in LMICs. Nonetheless, any center providing pediatric care must always have staff with expertise in pediatric resuscitation available. In addition, reciprocal relationships need to be in place for the provision of a higher level of care if transfer is deemed necessary.^12^

The development of partnerships between LMIC-LMIC and HIC-LMIC gave rise to the Global Initiative for Children's Surgery (GICS), responsible for the identification of the priorities for surgical children and the development of guidelines for Optimal Resources for Children's Surgery (OReCS).^13^ Based on their recommendations, first-level hospitals have the infrastructure and workforce to admit healthy children for common surgical procedures, resuscitation, and emergency surgical care. Sicker children and/ or younger than 1 year old requiring more complicated surgical procedures should receive perioperative treatment in higher levels of care unless emergent. The most experienced available anesthesia providers should be in charge. Secondary and tertiary-level hospitals must have specialists with pediatric experience in anesthesia for the comprehensive care of more complex cases and children with comorbidities. These recommendations are aligned with the World Federation of Societies of WFSA and the Association of Anaesthetists to enhance the quality of children's care by providing training in LMICs.

Programs such as SAFE Paeds – a branch of the Safer Anaesthesia Education (SAFE) initiative have led to improvement in the knowledge, skills, and behavior of anesthesia providers. Opportunities for advanced training in pediatric anesthesia to develop future pediatric anesthesia leaders and educators should be encouraged. The goal is to guarantee that any hospital admitting a pediatric patient has a team of anesthesia providers led by at least one anesthesiologist appropriately trained.^8^ Academic training centers, mostly represented by tertiary referral centers, should be staffed with experienced pediatric anesthetists to ensure the quality of education training.

## Where and who

Unfortunately, in Brazil pediatric anesthesia encounters challenges based on the lack of either educational or financial incentives for healthcare professionals to pursue additional training. First, there is little or no movement of local staff from smaller institutions to seek additional training at specialized centers. Second, remuneration in pediatric anesthesia is shamefully inferior in comparison with other areas of anesthesia effectively placing the importance, needs, and well-being of children below most. It is important to notice that, most of the time, anesthesia practice occurs in the context of a mixed population varying from geriatric patients to infants in the same hospital on the same operating list. Children are commonly not pooled or cared for in a pediatric friendly environment.

It is well described that caseload and clinical experience are crucial to improve safety in pediatric anesthesia and the number of years of practice being the most common factor influencing the quality of care. Each year of anesthesia experience reduces 1% of respiratory events and 2% of cardiovascular events.^5^^,^^14^ The dedication of pediatric anesthesia performance of less than 73 days per year is an independent risk factor for the number of cardiac arrests in the operating and postoperative recovery room.^14^ In this way, not only the lack of adequate training but also the scarce maintenance of required skills exposes the children in need of anesthesia to suboptimal care in Brazil and in the majority of LMICs in South America.

In HICs, the economic pressure, time constraints, and demands of enhanced productivity conflict with the possibility of healthcare providers prioritizing education. This situation is even more challenging in LMIC.^15^ Data from the APRICOT study showed a variable incidence and management of severe perioperative critical events across Europe, raising concerns about pediatric anesthesia training, teams’ experience with higher-risk children, resources, and infrastructure.^5^ A sub‐analysis of the APRICOT, exploring the differences between Scandinavian data (Denmark, Finland, Norway, and Sweden) and the rest of Europe, indicated better outcome regarding the incidence and nature of perioperative critical events.^16^ This is not surprising given the unique practice and training in pediatric anesthesia in Scandinavia: the use of medications, anesthetic techniques, and training are uniform, there are always two anesthesia‐trained professionals, and in the pediatric recovery room, patients are accompanied by recovery‐trained and registered nurses. Aligned with this high standard of care, the Scandinavian countries have had a consistent training program in pediatric anesthesia and intensive care running for over 20 years. There is no doubt that all these factors continue to result in a safer perioperative outcome.^17^

## Future directions

Without changes in the investment in extra years of specific pediatric anesthesia training, either by providing financial funding or by standardizing and regulating pediatric anesthesia competencies, the current mixed anesthesia system will continue to disadvantage children in Brazil. In addition to organizing local pediatric specialized care, administrative strategies are necessary to guarantee and optimize the delivery of excellent care. The creation of specific guidelines and flowsheets related to referral criteria are essential, permitting objective decision making. Such strategies should be based on hospital infrastructure and equipment, population age, urgency of the procedure, complexity of anesthesia, postoperative care needs, pain management availability, and the experience of the anesthesia, surgical, and nursing teams involved.

Technological and pharmacological advances in anesthesia play an important role in the reduction of morbidity and mortality in perioperative settings worldwide. Adequate and well-functioning equipment is an elementary requirement for safe anesthesia in children and is extensively described by the World Federation of Societies of Anesthesiologists (WFSA) and the World Health Organization (WHO) Safe Surgery Checklist.^18^ Even though access to new technologies is variable in LMICs, improvement of care should not only focus on acquiring equipment but guaranteeing the proper use of the basic equipment by training personnel and maintenance of competence.^15^ Gaps in the preparation of appropriately sized pediatric equipment and monitors remain a major obstacle despite the availability of perioperative pediatric anesthesia equipment.^19^

Investing in infrastructure alone will not solve these challenges, developing an institutional competence does.^20^ In the pre-pandemic era, the Global Initiative for Children's Surgery stated that the delivery of safe, effective surgical care to children was already critical and neglected. The pandemic has aggravated this scenario posing unprecedented challenges to the healthcare system worldwide. Even though the adult population bore the brunt of the pandemic as reflected in the number of deaths, the impact on the pediatric population cannot be underestimated.^21^ The fear of reaching the hospitals resulted in diagnostic as well as therapeutic delays and preventable complications.^22^

Longer surgical waiting lists and diseases that are more advanced have an even bigger impact on pediatric anesthesia practice, exacerbating the existing surgical backlogs and delays for elective, urgent, and emergent cases.^23^ Brazil and other South American LMICs need urgent mobilization of resources and focus on strategies to mitigate the already overwhelmed healthcare provision for children. The exponential growth in pediatric surgical need will increase the ratio between mixed-practice anesthetists and pediatric patients. Nonetheless, special efforts must be made to provide long-term strategies to guarantee pediatric anesthesia care.

The morbidity and mortality in the pediatric population undergoing surgery can be significantly improved by implementing simple strategies. As anesthetists, we should ask ourselves uncomfortable questions: Are we able to provide appropriate care for these children needing surgery? and When will we be ready to put safety into practice? In addition, most importantly: Are we ready to respect the right of the child to enjoy the highest attainable standard of health?

## Declaration of Competing Interest

The authors declare no conflicts of interest.
